# Diuretics in Cardiovascular Disease: For How Long and at What Dose?

**DOI:** 10.3390/jcdd13080395

**Published:** 2026-08-17

**Authors:** Ioannis Paraskevaidis, Elias Tsougos, Christos Kourek

**Affiliations:** 1 Medical School of Athens, National and Kapodistrian University of Athens, 15772 Athens, Greece; iparas@otenet.gr; 2 Department of Cardiology, Hygeia Hospital, 15123 Athens, Greece; tsougos@yahoo.com

**Keywords:** heart failure, congestion, diuretic resistance, loop diuretics, thiazide diuretics, mineralocorticoid receptor antagonists, electrolyte imbalance, chronic kidney disease, elderly, deprescribing

## Abstract

Diuretics remain indispensable for the treatment of congestion in cardiovascular disease, particularly in acute and chronic heart failure. Their principal clinical value is rapid symptom relief, reduction in filling pressures and facilitation of decongestion; however, a mortality benefit has not been consistently demonstrated. This distinction is clinically important because long-term diuretic exposure in patients without objective congestion may promote hypotension, renal dysfunction, electrolyte instability, neurohormonal activation, falls, cognitive impairment and avoidable polypharmacy. The challenge is therefore not whether diuretics should be used, but how intensively, for how long and under which monitoring strategy. In this narrative review, we summarize the pharmacology of major diuretic classes, the mechanisms of diuretic resistance, and the clinical consequences of potassium, sodium and magnesium disorders. We also discuss drug interactions, frailty, chronic kidney disease, pregnancy, heart failure with preserved ejection fraction and the emerging role of sodium–glucose cotransporter 2 inhibitors and adjunctive proximal-tubule strategies. A practical approach is proposed: use loop diuretics promptly when congestion is present, reassess response with symptoms, weight, urine output, renal function, electrolytes and congestion markers, and reduce or discontinue therapy once euvolemia is achieved and maintained. Deprescribing should be individualized, gradual and reversible, with explicit thresholds for restarting treatment. Optimized diuretic care requires the same discipline applied to disease-modifying heart failure therapy: phenotype recognition, dose minimization, close follow-up and avoidance of treatment inertia.

## 1. Introduction

Diuretics are among the most frequently prescribed drugs in cardiovascular medicine and remain the cornerstone of symptomatic treatment when central or peripheral congestion is present [1]. In acute decompensated heart failure, pulmonary edema or clinically overt volume overload, the indication is clear: prompt natriuresis and water removal improve dyspnea, reduce venous pressures and facilitate stabilization. The uncertainty begins after decongestion. Many patients are discharged or followed chronically on the same dose that was required during an unstable episode, even when physical examination, body weight, natriuretic peptides, renal function and functional status suggest a transition to euvolemia. In this setting, continued exposure may no longer be therapeutic; it may become a driver of hypovolemia, electrolyte imbalance, renal dysfunction and treatment complexity.

Current guidelines emphasize the importance of diuretics for relief of congestion, but they provide less operational guidance on dose reduction, discontinuation and reinitiation [2]. This is particularly relevant in older and frail patients, who are often underrepresented in trials yet carry the highest burden of multimorbidity, polypharmacy and adverse drug reactions [3,4]. Observational data have linked higher loop-diuretic exposure with worse outcomes in chronic heart failure, although confounding by disease severity remains a major limitation [5]. A more clinically useful question is therefore not whether diuretics are good or bad, but whether a given patient remains congested, which type of diuretic is most appropriate according to the clinical setting and the underlying mechanism of congestion or diuretic resistance, whether the dose is proportionate to the hemodynamic problem, and whether alternative strategies or lower doses can maintain stability. Loop diuretics remain the preferred agents when rapid and potent decongestion is required, whereas thiazide or thiazide-like diuretics may be added for sequential nephron blockade in patients with an inadequate response to loop therapy. In selected patients, proximal-tubule inhibition with acetazolamide or electrolyte-free water removal with vasopressin antagonists may also be considered according to the mechanism of congestion, renal function and electrolyte profile [6,7,8,9,10,11,12,13,14].

This review reframes diuretics as dynamic, phenotype-dependent therapy rather than indefinite background medication. We summarize mechanisms of action, adverse effects, electrolyte disorders, drug interactions, special populations and practical monitoring. The central thesis is that diuretics should be escalated decisively when congestion is present, but tapered or deprescribed when congestion has resolved, provided that surveillance is structured and the patient has clear instructions for early detection of recurrence [15,16,17].

The problem is intensified by the success of contemporary heart failure therapy. Patients increasingly receive angiotensin receptor–neprilysin inhibitors, beta-blockers, mineralocorticoid receptor antagonists and sodium–glucose cotransporter 2 inhibitors, each of which may influence blood pressure, renal hemodynamics or electrolyte balance. A loop-diuretic dose that was tolerated before optimization of disease-modifying therapy may become excessive once afterload, neurohormonal tone and glycosuria-related natriuresis change. Conversely, fear of renal deterioration may lead to underdiuresis in patients with true congestion, leaving elevated filling pressures untreated. High-quality care therefore requires repeated reconciliation between the hemodynamic phenotype and the pharmacologic regimen, rather than a static prescription copied from one clinical encounter to the next.

## 2. Pharmacologic Framework and Mechanisms of Action

Diuretics are classified by the nephron segment and transporter system on which they act. Carbonic anhydrase inhibitors such as acetazolamide reduce proximal tubular sodium and bicarbonate reabsorption, thereby increasing distal sodium delivery and urinary bicarbonate loss [6,7]. Loop diuretics, including furosemide and bumetanide, inhibit the sodium–potassium–chloride cotransporter in the thick ascending limb of Henle and are the most potent natriuretic agents used in cardiovascular practice. Thiazide and thiazide-like agents inhibit the sodium–chloride cotransporter in the distal convoluted tubule and are less potent as a monotherapy, but they are highly effective as a sequential nephron blockade when loop-diuretic response is inadequate [8,9].

Potassium-sparing diuretics act more distally. Mineralocorticoid receptor antagonists reduce aldosterone-mediated sodium reabsorption through epithelial sodium channels and limit potassium excretion; amiloride and related agents block the channel more directly. In heart failure, mineralocorticoid receptor antagonists are not merely potassium-sparing diuretics: they are disease-modifying therapies with antifibrotic and anti-inflammatory effects, but their use requires surveillance for hyperkalemia and worsening renal function [10,18,19,20,21,22]. Osmotic diuretics, such as mannitol, generate an osmotic gradient and are used mainly outside routine cardiovascular care because they may worsen intravascular expansion and pulmonary congestion. Vasopressin V2-receptor antagonists, commonly referred to as vaptans, antagonize the action of arginine vasopressin (antidiuretic hormone) at V2 receptors located on the basolateral membrane of principal cells in the late distal nephron, particularly the collecting duct. V2-receptor blockade reduces cyclic adenosine monophosphate-mediated trafficking and insertion of aquaporin-2 water channels into the apical membrane, thereby decreasing water reabsorption and promoting electrolyte-free water excretion (aquaresis). Tolvaptan, a selective oral V2-receptor antagonist, can therefore be useful in selected patients with euvolemic or hypervolemic hyponatremia, including patients with heart failure, although clinical trials have not demonstrated an improvement in long-term mortality or heart-failure-related morbidity [10,23,24].

Several drugs have diuretic-like properties without being conventional diuretics. Sodium–glucose cotransporter 2 inhibitors cause transient natriuresis and osmotic diuresis and have become foundational therapy across heart failure phenotypes. Their benefit appears to extend beyond fluid removal and includes renal, metabolic and myocardial effects [21,25]. Angiotensin-converting enzyme inhibitors and angiotensin receptor blockers may also exert mild natriuretic effects by reducing renin–angiotensin–aldosterone system activity, but hypotension, renal impairment and electrolyte disorders can occur when they are combined with aggressive diuresis [26,27,28].

Pharmacokinetic differences among loop diuretics also influence clinical use. Furosemide has variable oral bioavailability and a relatively short half-life, whereas bumetanide and torsemide have more predictable absorption and longer duration of action in many patients [11,12]. These differences become clinically relevant in intestinal edema, advanced kidney disease, unstable oral intake or recurrent post-discharge congestion. However, switching agents should not replace reassessment of the patient. Apparent failure of furosemide may reflect poor absorption, but it may also reflect inadequate dose, excessive dietary sodium, advanced right-sided congestion, non-adherence or distal nephron adaptation. The choice of drug, route and schedule should therefore be linked to the suspected mechanism of poor response.

## 3. Diuretic Resistance and Determinants of Response

The response to a diuretic dose is not fixed. It is influenced by intestinal absorption, plasma protein binding, renal perfusion, albumin concentration, venous congestion, tubular adaptation, neurohormonal activation and patient adherence [6,7,8]. Loop diuretics must reach the tubular lumen to act; intestinal edema may reduce oral absorption, low albumin may impair delivery, and elevated renal venous pressure may reduce filtration and tubular secretion. These factors explain why a patient with right-sided congestion or cardiorenal syndrome may appear diuretic-resistant despite receiving an apparently adequate dose.

Chronic loop-diuretic therapy can reduce subsequent natriuretic responsiveness through the so-called “*braking phenomenon*”. This phenomenon reflects a progressive adaptation to repeated diuretic exposure, including hypertrophy and hyperplasia of distal tubular epithelial cells, increased sodium reabsorption downstream of the loop of Henle, and activation of neurohormonal pathways that promote sodium avidity [13]. Consequently, the same loop-diuretic dose may produce progressively less natriuresis over time. The “*braking phenomenon*” should be distinguished from post-diuretic sodium retention, which occurs after the natriuretic effect of an individual loop-diuretic dose has subsided. As the tubular concentration of the drug falls below its effective threshold, compensatory sodium reabsorption increases during the interdose period and may partially offset the sodium loss achieved during the preceding period of natriuresis [1]. Post-diuretic sodium retention is therefore primarily a short-term phenomenon related to the limited duration of loop-diuretic action and the interval between doses, whereas the “*braking phenomenon*” reflects longer-term renal adaptation to repeated diuretic exposure. These mechanisms may coexist and contribute to an inadequate net natriuretic response, but they have different therapeutic implications: shortening the interdose interval may limit post-diuretic sodium retention, whereas chronic distal nephron adaptation may require sequential nephron blockade rather than continued escalation of loop-diuretic dose alone [1,13].

Diuretic resistance should be distinguished from absence of congestion. In the former, the patient remains volume-overloaded and requires a better decongestive strategy. In the latter, persistent diuretic administration exposes the patient to avoidable harm. This distinction is difficult when congestion is predominantly splanchnic, renal or interstitial rather than peripheral. A structured assessment should combine symptoms, orthopnea, edema, jugular venous pressure, lung and abdominal findings, body weight trajectory, urine output, natriuretic peptides, serum creatinine, blood urea nitrogen, chloride and albumin. No single marker is sufficient, but discordant signals should prompt reassessment rather than automatic dose escalation [29,30,31,32,33,34,35,36,37].

A useful bedside distinction is between pharmacokinetic resistance, in which the drug fails to reach the nephron in sufficient concentration, and pharmacodynamic resistance, in which the nephron compensates despite adequate drug delivery. The first pattern may respond to intravenous administration, an adequate increase in loop-diuretic dose, a change in loop diuretic, or correction of factors impairing drug delivery, whereas the second is more likely to require sequential nephron blockade and treatment of the mechanisms promoting renal sodium avidity [12]. Early assessment of post-diuretic natriuresis provides an objective method for evaluating this response before relying on changes in body weight or symptoms. A spot urinary sodium concentration obtained approximately 1–2 h after intravenous loop-diuretic administration can identify an inadequate natriuretic response; values below approximately 50–70 mmol/L have commonly been proposed as indicating insufficient response and should prompt reassessment of dose, route, renal function and ongoing congestion rather than automatic continuation of the same regimen [12,38]. Importantly, a low post-diuretic urinary sodium concentration alone does not establish true diuretic resistance: an initially inadequate dose or impaired drug delivery may produce the same finding. Persistent poor natriuresis despite appropriately escalated loop-diuretic exposure is more suggestive of diuretic refractoriness and may justify sequential nephron blockade [12].

More quantitative assessment can be obtained with the natriuretic response prediction equation (NRPE), which uses an approximately 2 h post-diuretic spot urine sample, incorporating urinary sodium and creatinine together with renal function and body size, to estimate cumulative natriuresis over the subsequent hours [39]. The NRPE has shown high accuracy for identifying poor loop-diuretic natriuretic response and may overcome an important limitation of urinary sodium concentration alone, namely, that the latter does not account for urine concentration [39]. Early natriuretic response also carries prognostic information: lower sodium excretion after loop-diuretic administration has been associated with less effective decongestion, longer hospitalization and worse subsequent clinical outcomes [40,41]. Furthermore, urinary sodium can be used not only for risk assessment but also to guide therapy. In the randomized PUSH-AHF trial, a natriuresis-guided strategy using serial spot urinary sodium measurements, with treatment intensification when urinary sodium was <70 mmol/L and/or diuresis was inadequate, increased 24 h natriuresis and diuresis compared with standard care without an excess of renal safety events, although it did not reduce 180-day mortality or heart failure rehospitalization [42]. Thus, post-loop-diuretic natriuresis is best considered an early, actionable marker of pharmacological response that complements urine output, renal function, congestion assessment and clinical trajectory rather than a stand-alone definition of diuretic resistance [12,42].

**Figure 1:**
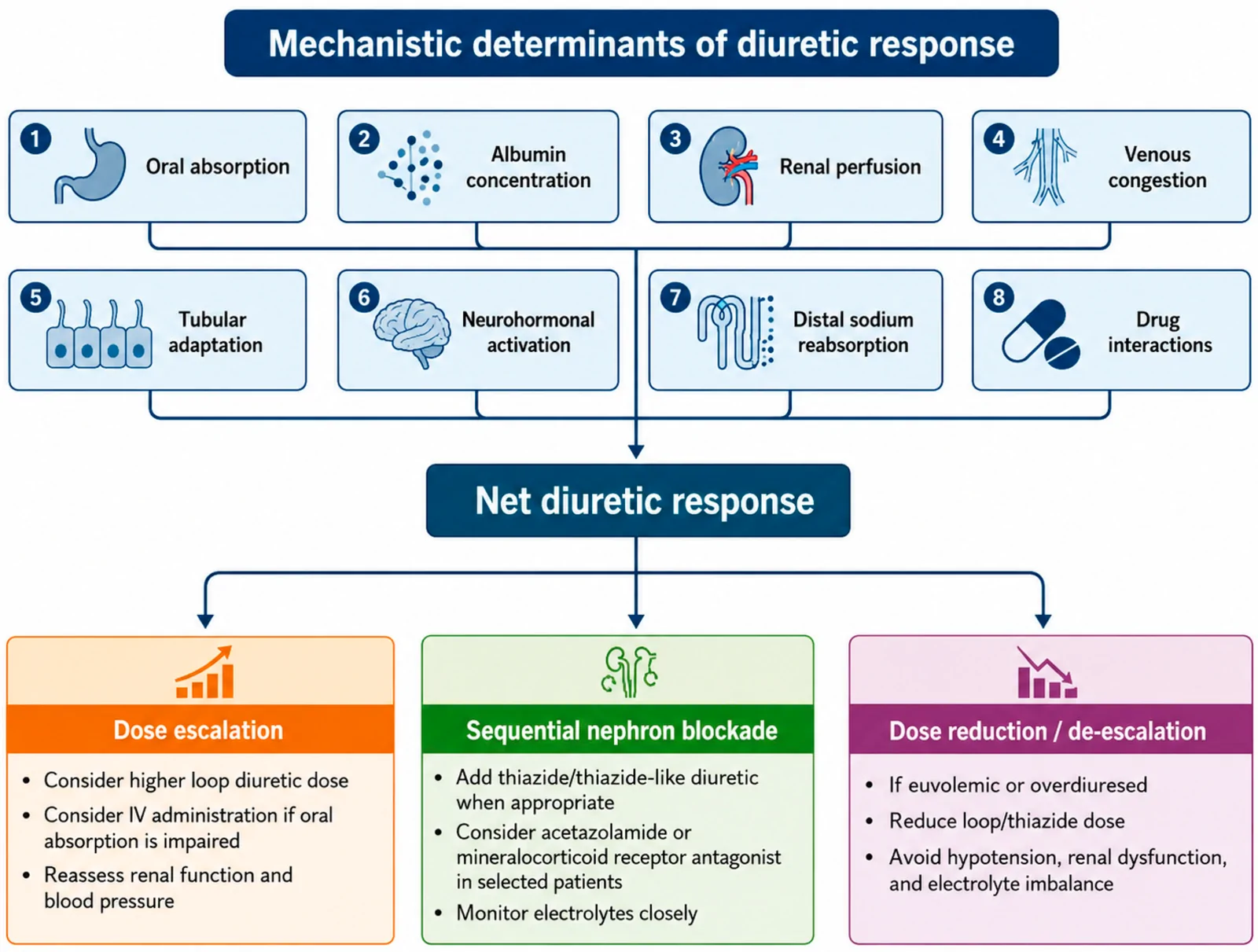
Mechanistic determinants of diuretic response. Oral absorption, albumin concentration, renal perfusion, venous congestion, tubular adaptation, neurohormonal activation, distal sodium reabsorption and drug interactions determine whether a patient requires dose escalation, sequential nephron blockade or dose reduction.

Early urine output, spot urinary sodium after loop-diuretic administration, net fluid balance, weight trajectory and symptoms should be interpreted together, not in isolation (Figure 1).

## 4. Electrolyte Imbalance and Arrhythmic Vulnerability

Electrolyte abnormalities are common in heart failure and are strongly shaped by diuretic therapy, renal dysfunction, neurohormonal activation, diet, cachexia and concomitant medications. Disturbances of potassium, sodium, magnesium and phosphate are clinically important because they may worsen symptoms, limit disease-modifying therapy and increase arrhythmic risk [43]. Potassium deserves special attention. Hypokalemia increases myocardial excitability and pacemaker automaticity, whereas hyperkalemia depresses automaticity and conduction. Both extremes may provide an arrhythmogenic substrate, especially in patients with structural heart disease, renal impairment or concomitant renin–angiotensin–aldosterone system inhibition [44,45,46]. A practical target is to avoid both depletion and excess, with closer monitoring after dose changes, intercurrent illness or addition of mineralocorticoid receptor antagonists, angiotensin receptor–neprilysin inhibitors or sodium–glucose cotransporter 2 inhibitors. Intercurrent illnesses associated with gastrointestinal fluid losses, even mild diarrhea or vomiting, may precipitate volume depletion, electrolyte disturbances and worsening renal function in patients receiving diuretics. Particular caution is warranted in vulnerable populations, especially older adults and patients with chronic kidney disease, in whom clinical status, renal function and electrolytes should be reassessed and temporary adjustment of the diuretic dose considered when appropriate [47].

Hyponatremia affects 20–30% of patients with heart failure and is associated with fatigue, nausea, confusion, longer hospitalization and increased mortality [43]. Dilutional hyponatremia reflects water retention mediated by vasopressin and neurohormonal activation, whereas depletional hyponatremia is more often related to salt restriction, gastrointestinal losses or excessive diuretic therapy. The distinction matters: intensifying loop diuretics in depletional hyponatremia may worsen hypotension, renal function and electrolyte instability. Clinical signs, urine osmolality, urinary sodium and the overall volume profile should guide management, although interpretation is imperfect in patients already receiving diuretics.

Magnesium depletion is also common and clinically under-recognized. Loop and thiazide diuretics increase urinary magnesium loss, and hypokalemia may further impair magnesium reabsorption [48,49,50]. Low magnesium can prolong repolarization, promote QT prolongation and facilitate ventricular arrhythmias, particularly when combined with hypokalemia or other proarrhythmic drugs [51]. Magnesium status should therefore be assessed when diuretic doses are high, potassium is difficult to correct, renal function fluctuates, or unexplained ectopy and QT prolongation occur. The broader point is that electrolyte surveillance should not be regarded merely as a routine protocol; it is central to safe diuretic prescribing.

Chloride is often neglected despite its relevance to diuretic response and acid-base status. Loop and thiazide diuretics may produce hypochloremic metabolic alkalosis, which can reduce natriuretic efficiency and reinforce neurohormonal sodium retention. In advanced heart failure, low chloride may therefore be both a marker and a mediator of diuretic resistance. Proximal-tubule strategies such as acetazolamide are attractive partly because they counter bicarbonate retention and may restore a more favourable tubular environment for natriuresis [13,14,52]. Clinically, a patient with rising bicarbonate, falling chloride, hypokalemia and persistent congestion should not simply receive more loop diuretic; the electrolyte pattern should influence the next step.

## 5. Drug Interactions, Polypharmacy and the Older Patient

Diuretics are rarely used in isolation. Beneficial combinations include loop or thiazide diuretics with potassium-sparing agents when hypokalemia is a limiting problem. Potentially harmful combinations are equally common. Non-steroidal anti-inflammatory drugs blunt renal prostaglandin-mediated vasodilation and may reduce natriuretic response while worsening renal function. Corticosteroids may promote sodium retention and potassium loss. Loop or thiazide therapy combined with renin–angiotensin–aldosterone system inhibitors may stabilize potassium in some patients but may also precipitate hypotension, hyponatremia or renal dysfunction when volume depletion is present. Thiazides may worsen glucose and uric-acid control, and their combination with beta-blockers can aggravate metabolic adverse effects in susceptible patients [26,28].

Older adults require a different threshold for concern. Ageing is associated with lower glomerular filtration rate, reduced muscle mass, altered drug distribution, impaired thirst regulation, autonomic dysfunction and higher susceptibility to orthostatic hypotension. Multimorbidity and polypharmacy amplify risk [53]. In chronic stable heart failure, higher loop-diuretic doses may interfere with optimization of disease-modifying therapies and are associated with worse outcomes and lower tolerability, particularly when congestion is absent or minimal [54,55,56,57]. Adverse drug events are a major cause of hospitalization in older adults, and many are preventable [58,59,60,61].

Deprescribing is therefore not therapeutic nihilism; it is active risk management. Evidence syntheses suggest that diuretic reduction or discontinuation can be feasible in selected stable patients, although protocols and outcome data remain heterogeneous [15,17]. The appropriate candidate is typically euvolemic, hemodynamically stable, adherent to follow-up and able to monitor symptoms and body weight. The inappropriate candidate is a patient with unresolved congestion, recent recurrent decompensation, severe right-sided failure, advanced renal dysfunction with sodium avidity, or inability to access timely reassessment. The same patient may move between categories over time, which is why diuretic therapy should be reviewed repeatedly rather than treated as permanent [62,63,64].

Frailty adds a functional dimension that is not captured by creatinine or ejection fraction. A small absolute fall in blood pressure or sodium may be well tolerated by a robust younger patient but may precipitate delirium, falls or loss of independence in an older person with sarcopenia and impaired balance. Serum creatinine may underestimate the degree of renal impairment in patients with reduced muscle mass, resulting in overestimation of kidney function by creatinine-based estimated glomerular filtration rate (eGFR). In such patients, measurement of cystatin C, either alone or preferably in combination with creatinine using validated eGFR equations, can provide a more informative assessment of kidney function. Cystatin C also has prognostic relevance in heart failure and cardiorenal syndrome, with elevated concentrations and worsening cystatin C-based renal function being associated with adverse clinical outcomes [47,65,66]. For this reason, the monitoring plan in older adults should include orthostatic symptoms, standing blood pressure when feasible, appetite, fluid intake, cognition, fall history and caregiver capacity. The threshold for reducing a chronic diuretic dose should be lower when congestion is absent and functional harms are emerging.

## 6. Clinical Indications and Dose Optimization

The indication for diuretic therapy differs across clinical scenarios. In acute heart failure with pulmonary congestion, intravenous loop diuretics remain first-line therapy, with dose guided by prior exposure, renal function, urine output, natriuresis and clinical trajectory [11,12,33]. Persistent congestion at discharge is associated with worse outcomes; premature discontinuation is therefore unsafe when objective overload remains. Conversely, continuing high-dose loop therapy after decongestion may be harmful and has been associated with worse clinical and patient-reported outcomes after hospitalization [11]. The therapeutic goal is complete but not excessive decongestion.

When loop response is inadequate, several adjunctive strategies can be considered. Thiazide-type diuretics provide sequential nephron blockade and are particularly useful when distal sodium reabsorption is increased, but they require close monitoring for hyponatremia, hypokalemia, hypomagnesemia and renal dysfunction [67,68,69]. Acetazolamide targets proximal sodium and bicarbonate reabsorption and can enhance decongestion when added to loop diuretics in acute decompensated heart failure with volume overload [13,14,52]. Vasopressin antagonists increase electrolyte-free water excretion and may be useful in selected patients with hyponatremia; however, short-term symptomatic improvement has not translated into robust mortality benefit [23,70].

Sodium–glucose cotransporter 2 inhibitors occupy a distinct position. They can increase urine output and weight loss in hospitalized and ambulatory heart failure populations while exerting modest effects on electrolyte balance and renal hemodynamics compared with conventional diuretics [71,72,73,74,75]. They should not be viewed simply as weak diuretics, because their outcome benefits are broader and persist beyond their early natriuretic phase, providing cardiovascular and renal protection through reductions in worsening heart failure and heart failure hospitalization and by slowing the progression of chronic kidney disease, with benefits observed across a broad range of left ventricular ejection fraction and irrespective of the presence of diabetes in several studied populations [76,77]. Nevertheless, when sodium–glucose cotransporter 2 inhibitors are initiated in patients receiving high-dose loop diuretics, clinicians should reassess volume status to avoid hypotension or overdiuresis, especially in older adults and those with low baseline blood pressure.

Right-sided heart failure deserves separate consideration because venous congestion may impair renal function even when left-sided pulmonary signs are modest. Elevated renal venous pressure reduces the transrenal perfusion gradient and promotes sodium retention, creating a vicious cycle of congestion, renal dysfunction and reduced diuretic delivery. Clinicians may respond by increasing diuretic doses, but excessive intravascular depletion can worsen forward flow and renal perfusion. The therapeutic target is relief of venous congestion while preserving effective arterial volume. This often requires smaller, repeated adjustments, careful monitoring of renal indices and consideration of the underlying driver, such as pulmonary hypertension, severe tricuspid regurgitation, constrictive physiology or right ventricular failure.

## 7. Congestion Assessment and Monitoring

The safest diuretic strategy begins with defining congestion as accurately as possible. Weight change and urine output are useful but incomplete. Clinical congestion scores incorporating edema, orthopnea, rales, ascites and pleural effusion may standardize assessment, but validation is limited and interobserver variability remains important [29,30,31,32]. Jugular venous pressure provides valuable information, particularly when elevated above conventional thresholds, but bedside assessment is technically challenging and may be inaccurate. Ultrasound and near-infrared spectroscopy have been explored as non-invasive approaches to estimate right atrial pressure and venous congestion, but their routine role requires further validation [34,35,36].

Biochemical and hemodynamic markers may refine decision-making. Natriuretic peptides reflect myocardial wall stress but are influenced by age, rhythm, body mass index and renal function. Hematocrit, albumin and protein ratios may suggest hemoconcentration or oncotic factors, but they are not direct measures of decongestion [37]. Emerging biomarkers, including circulating mitochondrial DNA, are scientifically promising but remain investigational for routine diuretic titration [78,79,80]. Implantable pulmonary artery pressure monitoring can support hemodynamic-guided care in selected patients, although the optimal implementation strategy is still evolving [81].

A pragmatic monitoring plan should be simple enough for daily practice: reassess symptoms, edema, orthopnea, blood pressure, heart rate, body weight, renal function, sodium, potassium, magnesium and chloride after initiation or dose change; review concomitant nephrotoxic or hypotensive drugs; and define an action threshold for recurrence of congestion. If euvolemia is maintained, gradual reduction to the lowest effective dose is reasonable. If congestion recurs, the previous effective dose can be restored and the precipitating factor should be sought. This reversible approach is safer than both indefinite high-dose exposure and abrupt discontinuation without surveillance.

The limitations of individual clinical and biochemical markers support a multimodal approach to congestion assessment. Bioelectrical impedance analysis (BIA) is a rapid, non-invasive technique that evaluates body fluid status by measuring tissue opposition to a low-amplitude electrical current. Bioimpedance-derived indices can provide information on total and extracellular fluid burden and may identify residual or subclinical congestion that is not apparent on physical examination. In patients with heart failure, bioelectrical impedance vector analysis has demonstrated additional diagnostic and prognostic value when combined with conventional clinical and biochemical markers, although measurements may be influenced by body composition, device characteristics and methodological differences and should therefore be interpreted within the overall clinical context [82,83]. Lung ultrasound (LUS) provides a complementary bedside assessment of pulmonary congestion: the presence and number of B-lines reflect increased extravascular lung water, while ultrasonography can also readily detect pleural effusions. Serial assessment of B-lines can identify residual pulmonary congestion and monitor changes during decongestive therapy, and randomized studies suggest that LUS-guided management may reduce episodes requiring urgent treatment for worsening heart failure [84,85]. Finally, the Venous Excess Ultrasound (VExUS) score has emerged as a point-of-care method for evaluating systemic venous congestion. VExUS integrates inferior vena cava assessment with Doppler flow patterns in the hepatic, portal and intrarenal veins to grade the severity of venous congestion [86]. Higher VExUS grades have been associated with adverse renal and heart-failure-related outcomes and may provide useful complementary information when titrating decongestive therapy, particularly in patients with right-sided or cardiorenal congestion [87]. However, further prospective interventional validation is required before VExUS can be considered a stand-alone target for diuretic titration. Taken together, BIA, LUS and VExUS provide complementary information on systemic volume status, pulmonary congestion and systemic venous congestion, respectively, and can strengthen clinical decision-making when integrated with symptoms, physical examination, renal function and biomarkers.

Post-discharge monitoring is the decisive phase for preventing both relapse and overtreatment. A patient discharged after acute heart failure should not remain indefinitely on the discharge diuretic dose without review. The first outpatient reassessment should determine whether the discharge dose is still needed, whether renal function and electrolytes have stabilized, and whether symptoms reflect congestion, low output, hypotension, deconditioning or comorbidity. Remote monitoring of body weight and symptoms can help, but it should be paired with actionable thresholds. For example, progressive weight gain with edema should trigger temporary intensification, whereas dizziness, low blood pressure, rising creatinine and stable or falling weight should prompt dose reduction and laboratory reassessment.

## 8. Special Clinical Situations

Chronic kidney disease is common in heart failure and modifies both risk and response [88]. Reduced glomerular filtration decreases diuretic delivery to the tubular lumen, often requiring higher loop-diuretic doses to achieve the same natriuretic effect. At the same time, renal dysfunction increases vulnerability to electrolyte disorders, hypotension and worsening kidney function. The natriuretic efficacy of conventional thiazide diuretics generally declines as kidney function deteriorates, and when eGFR falls below approximately 30 mL/min/1.73 m^2^, loop diuretics are usually preferred for the management of clinically significant fluid overload. This threshold should not, however, be regarded as absolute: thiazide-like agents such as chlorthalidone can retain clinically meaningful activity in advanced CKD, and thiazide or thiazide-like diuretics may be combined with loop diuretics as sequential nephron blockade in patients with persistent congestion or diuretic resistance [67,68,69]. Such strategies require close surveillance for hyponatremia, hypokalemia, volume depletion and worsening renal function.

Pregnancy with decompensated heart failure is uncommon but high-risk [89]. Diuretics may reduce maternal congestion, but excessive volume depletion can theoretically compromise uteroplacental perfusion. When necessary, cautious loop-diuretic use, attention to electrolytes and maternal hemodynamics, and multidisciplinary care are appropriate; mineralocorticoid receptor antagonists are generally avoided because of hormonal effects and safety concerns [90]. In the elderly, the main hazards are hypotension, falls, cognitive fluctuation, renal dysfunction and electrolyte instability. A meta-analysis has linked diuretic use with increased fall risk in older adults, reinforcing the need for dose minimization when congestion is absent [91].

Heart failure with preserved ejection fraction illustrates why phenotype matters. In many patients, symptoms reflect interstitial fluid redistribution, vascular stiffness, obesity, atrial fibrillation, pulmonary hypertension and renal impairment rather than simple intravascular excess. Diuretics remain appropriate for overt congestion, but high doses can reduce preload excessively and worsen renal function or exercise tolerance. The benefits of sodium–glucose cotransporter 2 inhibitors in heart failure with preserved or mildly reduced ejection fraction have changed the therapeutic landscape and should encourage more disciplined reassessment of chronic loop-diuretic need [92,93,94,95].

Hypoalbuminemia and non-cardiac edema illustrate the limits of reflexive diuretic escalation. Low oncotic pressure can produce edema even when intravascular volume is not expanded; in such cases, high-dose diuretics may reduce effective circulating volume without resolving the underlying mechanism. In heart failure, the interpretation is more complex because low albumin may coexist with venous congestion, inflammation, malnutrition and endothelial dysfunction. The key clinical task is to determine whether the edema is primarily hydrostatic, oncotic, inflammatory or mixed. Treatment may require nutritional assessment, management of renal or hepatic disease, adjustment of vasodilators or venous pressures, and only then diuretic titration.

## 9. Practical Approach to Tapering and Deprescribing

**Figure 2:**
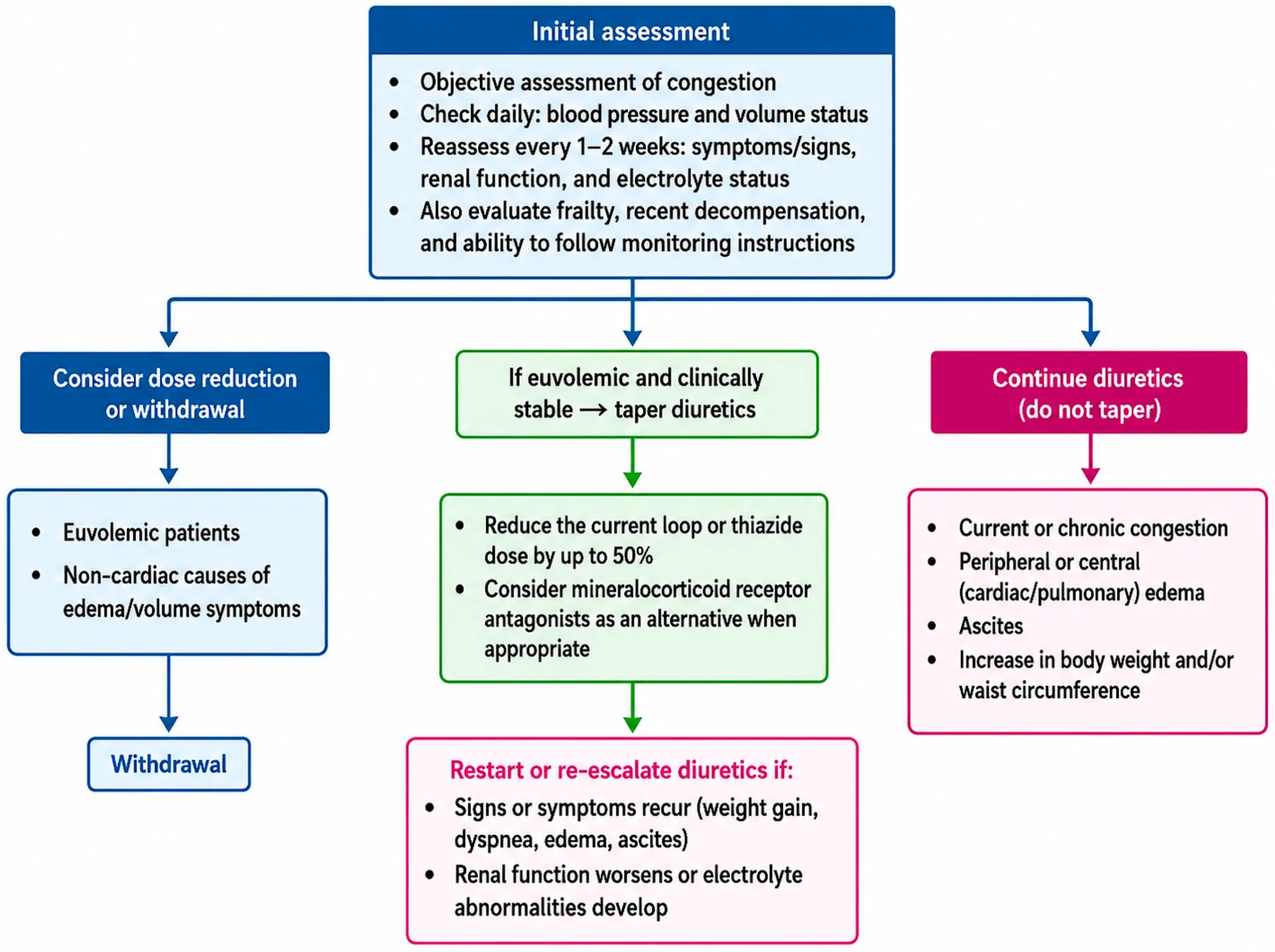
Proposed decision pathway for diuretic continuation, tapering or deprescribing in patients with cardiovascular disease. The pathway begins with objective assessment of congestion, followed by evaluation of renal function, electrolytes, blood pressure, frailty, recent decompensation and ability to follow monitoring instructions.

A practical deprescribing algorithm should start with a positive question: what evidence shows that this patient still needs the current diuretic dose? If the patient has no edema, no orthopnea, stable weight, acceptable natriuretic peptide trend, stable renal function and no recent congestion-driven hospitalization, dose reduction can be considered. The first step is usually a partial reduction rather than abrupt cessation, particularly after recent decompensation or in patients with right-sided heart failure. Follow-up should include a planned clinical or remote review, repeat laboratory testing when risk is high, and explicit patient instructions for weight gain, dyspnea, edema, dizziness or hypotension (Figure 2).

Restarting diuretics should be equally structured. A short-term increase may be appropriate for rapid weight gain, recurrent edema or worsening dyspnea, but persistent need for repeated rescue doses should trigger reassessment for dietary sodium excess, non-adherence, renal dysfunction, atrial fibrillation, infection, drug interactions or progression of heart failure. Clinicians should also consider whether disease-modifying therapy has been optimized. High loop-diuretic requirement is often a marker of residual congestion or advanced disease; it should prompt a search for cause rather than passive continuation.

A written plan improves safety. The plan should specify the current target weight or weight range, symptoms that suggest recurrent congestion, symptoms that suggest overdiuresis, the laboratory interval, and the exact dose to resume if congestion returns. Patients should be told that deprescribing is not permanent withdrawal of care; it is a monitored trial of the lowest effective dose. This framing reduces anxiety and improves adherence because the patient understands that treatment can be restarted promptly. Clinicians should document the reason for reduction, the follow-up date and the restart criteria so that other providers do not automatically reinstate high-dose therapy without reassessing volume status.

The evidence base for loop-diuretic de-escalation is considerably smaller and more heterogeneous than that supporting their use for active congestion, and the results of withdrawal studies need to be interpreted in the context of the treatment era and patient selection. Earlier studies conducted before contemporary guideline-directed medical therapy highlighted the potential risk of indiscriminate withdrawal. In a small randomized crossover study by Richardson et al., 4 of 14 patients with mild heart failure deteriorated when managed with captopril without diuretic therapy, developing pulmonary edema or worsening breathlessness; importantly, all four had a previous history of pulmonary edema [96]. Similarly, Grinstead et al. discontinued chronic diuretic therapy in 41 clinically stable patients with heart failure, of whom 29 (71%) required reinitiation of diuretics during 12 weeks of follow-up, with a median time to reintroduction of 15 days [97]. A baseline furosemide dose >40 mg/day, LVEF ≤ 27% and a history of systemic hypertension were associated with a greater likelihood of requiring diuretic reinitiation. Conversely, subsequent studies suggested that de-escalation may be feasible in appropriately selected patients. Galve et al. reported that 17 of 26 (65%) stable patients with systolic heart failure tolerated diuretic withdrawal for 3 months without deterioration in exercise capacity or NYHA functional class, although atrial natriuretic peptide concentrations increased [98]. More recently, the multicentre, double-blind ReBIC-1 trial randomized 188 stable outpatients with mild heart failure, no evidence of fluid retention and low-dose furosemide therapy to withdrawal or continued treatment [99]. Furosemide withdrawal did not increase self-reported dyspnea, and 75.3% of patients assigned to withdrawal remained free from furosemide reuse compared with 83.7% receiving continued therapy, while heart-failure-related clinical events were infrequent in both groups [99]. These findings support a trial of loop-diuretic reduction or withdrawal in carefully selected, stable and euvolemic patients, but they should not be extrapolated to patients with recent or residual congestion, high diuretic requirements or advanced heart failure. Furthermore, even ReBIC-1 preceded widespread use of the complete contemporary HFrEF therapeutic framework, including SGLT2 inhibitors, and randomized evidence specifically evaluating diuretic withdrawal in patients receiving current comprehensive guideline-directed medical therapy remains limited. Accordingly, deprescribing should be regarded as an individualized and reversible strategy rather than a routine therapeutic objective.

Contemporary guideline-directed medical therapy may itself reduce the requirement for loop diuretics, providing an additional rationale for periodic reassessment rather than automatic continuation of a fixed dose. In a post hoc analysis of PARADIGM-HF, patients randomized to sacubitril/valsartan experienced more frequent loop-diuretic dose reductions and fewer dose increases than those receiving enalapril, with progressively lower net loop-diuretic use during follow-up [100]. This diuretic-sparing effect, however, does not appear uniform across heart failure phenotypes. In PARAGON-HF, sacubitril/valsartan was associated with a modest early reduction in new loop-diuretic initiation or intensification compared with valsartan, but differences in mean loop-diuretic dose and discontinuation were not sustained during longer-term follow-up. Similar observations have emerged with sodium–glucose cotransporter 2 inhibition [101]. In EMPEROR-Reduced, the cardiovascular benefits of empagliflozin were preserved irrespective of baseline diuretic use or dose, and treatment was associated with a reduced subsequent requirement for diuretic intensification [102]. In EMPEROR-Preserved, empagliflozin decreased the likelihood of diuretic dose escalation and increased the likelihood of de-escalation and discontinuation among patients receiving background diuretic therapy. These findings suggest that effective disease-modifying treatment may permit reduction in loop-diuretic burden in selected patients as hemodynamic and renal status improve. However, diuretic de-escalation should not be assumed to be uniformly benign or undertaken automatically after initiation of guideline-directed therapy. Reduction or withdrawal in patients with residual congestion, high baseline diuretic requirements, recent decompensation, significant right-sided failure or limited renal reserve may precipitate recurrent fluid retention and clinical deterioration. Conversely, failure to reassess the loop-diuretic dose after initiation of therapies with natriuretic or hemodynamic effects may contribute to volume depletion, hypotension or worsening renal function; notably, EMPEROR-Preserved identified a higher rate of volume-depletion events with empagliflozin among patients receiving concomitant diuretics [103]. Therefore, contemporary GDMT should be regarded as an opportunity to reassess rather than automatically reduce diuretic therapy, with any de-escalation guided by serial evaluation of congestion, blood pressure, renal function, electrolytes and symptoms.

Implementation is equally important. In many health systems, diuretic prescriptions are renewed automatically because they are perceived as low risk and because congestion status is not documented with enough precision. A high-impact clinical message is that every diuretic prescription should have an indication, a target, a monitoring plan and a stop-or-reduce criterion. This approach mirrors antimicrobial stewardship: the drug is essential when indicated, but duration and reassessment determine safety. Embedding such prompts into discharge summaries, heart failure clinics and primary-care medication reviews could reduce preventable adverse events without increasing congestion-related admissions.

## 10. Conclusions

Diuretic management should be integrated into the broader architecture of cardiovascular care. The same patient may require aggressive intravenous decongestion during hospitalization, a moderate oral dose during early recovery and no chronic diuretic once disease-modifying therapy has stabilized the syndrome. This time-dependent trajectory should be anticipated rather than discovered through adverse events. Clinicians should therefore reassess diuretic need at every transition of care, after every major change in heart failure therapy, after intercurrent illness and whenever renal function, blood pressure or electrolytes shift.

Diuretics are essential for the relief of congestion in cardiovascular disease, but their benefit is conditional on the presence of fluid overload and the adequacy of monitoring. Overuse can promote hypovolemia, hypotension, renal dysfunction, electrolyte imbalance, arrhythmia, falls and avoidable polypharmacy, especially in frail older patients. Underuse, premature withdrawal or failure to overcome diuretic resistance can leave patients congested and vulnerable to rehospitalization and death. The correct strategy is therefore neither reflexive continuation nor indiscriminate deprescribing, but repeated phenotyping. Loop diuretics should be used promptly and effectively when congestion is present; adjunctive agents should be selected according to the mechanism of poor response; and therapy should be tapered to the lowest effective dose once euvolemia is achieved. Future studies should test standardized congestion assessment, deprescribing protocols and patient-centred monitoring pathways so that diuretics can be used with the same precision expected of contemporary cardiovascular therapeutics.

## Data Availability

No new data were created or analyzed in this study. Data sharing is not applicable to this article.

## Abbreviations

The following abbreviations are used in this manuscript:

BIA	Bioelectrical Impedance Analysis
CKD	Chronic Kidney Disease
eGFR	Estimated Glomerular Filtration Rate
GDMT	Guideline-Directed Medical Therapy
HFrEF	Heart Failure with Reduced Ejection Fraction
LUS	Lung Ultrasound
LVEF	Left Ventricular Ejection Fraction
NRPE	Natriuretic Response Prediction Equation
NYHA	New York Heart Association
SGLT2	Sodium–Glucose Co-Transporter 2
VExUS	Venous Excess Ultrasound
